# Stromal fibronectin expression in patients with resected pancreatic ductal adenocarcinoma

**DOI:** 10.1186/s12957-019-1574-z

**Published:** 2019-02-08

**Authors:** Dingyuan Hu, Daniel Ansari, Qimin Zhou, Agata Sasor, Katarzyna Said Hilmersson, Roland Andersson

**Affiliations:** 1 Department of Gastroenterology, The Second Affiliated Hospital and Yuying Children’s Hospital of Wenzhou Medical University, 109 Xueyuan West Road, Wenzhou, 325000 China; 2 Department of Surgery, Clinical Sciences Lund, Lund University and Skåne University Hospital, Getingevägen 4, SE-221 85 Lund, Sweden; 3 Department of Pathology, Skåne University Hospital, Getingevägen 4, SE-221 85 Lund, Sweden

**Keywords:** Fibronectin, Pancreatic ductal adenocarcinoma, Survival, Immunohistochemistry

## Abstract

**Background:**

Pancreatic ductal adenocarcinoma (PDAC) is characterized by an extremely dense stroma, which has a fundamental role in tumor progression. Fibronectin (FN1) is the main constituent of the tumor stroma in pancreatic cancer. This study aimed to explore the association between FN1 and clinicopathological characteristics and disease survival.

**Methods:**

Formalin-fixed paraffin-embedded tissue samples from 138 patients with PDAC were constructed into a tissue microarray, followed by immunohistochemical analysis with a recombinant monoclonal FN1 antibody. Chi-square test or Fisher’s exact test were used for comparison of FN1 expression and relevant clinicopathological parameters. Kaplan-Meier survival curves and Cox regression analyses were used to assess the association between FN1 and survival.

**Results:**

FN1 was detected in the stromal compartment in most cases (117/138, 84.8%). Compared to the low FN1 expression group, the high FN1 expression group had significantly larger tumor size (*P* = 0.002), more advanced T stage (*P* = 0.039) and N stage (*P =* 0.009), and also worse AJCC stage (*P* = 0.003). However, stromal FN1 expression was not associated with disease-free survival or overall survival.

**Conclusions:**

This study suggests that high stromal FN1 expression is associated with aggressive tumor characteristics in patients with resected PDAC. However, no association between FN1 expression and survival was found.

## Background

Pancreatic ductal adenocarcinoma (PDAC) is the third leading cause of cancer death, characterized by frequent metastases and profound chemoresistance [1]. The median survival for all stages of pancreatic cancer combined is 6 months, with a 5-year survival rate of less than 7% [2]. It is projected that PDAC will become the second cancer-related mortality within the next decade in the Western world [2]. During recent years, a marginal improvement in the treatment of PDAC has been seen, which can be exemplified by the ESPAC-4 clinical trial showing that gemcitabine-capecitabine combination therapy outperformed gemcitabine alone in patients with resected PDAC (median overall survival 28.0 vs 25.5 months) [3]. However, most therapeutic regimens for PDAC have failed, including antiangiogenetic approaches and immunotherapies, which have shown promise in, e.g., renal cell carcinoma and malignant melanoma [4]. It has been speculated that one major contributor to the treatment resistance is the hypovascular and immunosuppressive tumor microenvironment (TME), which is the most prominent histological feature of PDAC [5].

The TME in PDAC accounts for more than half of the tumor mass and has a complex role in tumor growth and the therapeutic response [5]. The high fibrotic stiffness of the TME compresses blood vessels and reduces perfusion that ultimately impedes the delivery of drugs to neoplastic cells. On the other hand, some constituents of tumor stroma act to suppress tumor growth by affecting the immune response and restraining tumor angiogenesis [6, 7]. A better characterization of TME is needed for more precise prediction of treatment response and development of new therapies.

Fibronectin (FN1) is a major constituent of the extracellular matrix within the TME and is not only produced mainly by fibroblasts, but also by tumor cells [8]. Normally, FN1 supports cell-ECM interactions and is essential for wound healing, development, and tissue homeostasis [9]. The binding of FN1 to its receptors, typically cell surface integrins, trigger FN1 signaling pathways in pancreatic tumor cells, promoting tumor cell survival and chemoresistance, cell invasion, metastasis, and angiogenesis [8]. Abrogating FN1-integrin interactions have produced strikingly positive pre-clinical results in various animal models of cancer by impeding angiogenesis and inhibiting tumor growth [10–12]. Unfortunately, however, these drugs, such as PF-04605412, have failed in clinical trials [13]. Further understanding of FN1 expression and function in the context of PDAC may potentially help to improve the effectiveness of FN1 inhibition in the clinical setting.

Immunohistochemical studies have confirmed that FN1 mainly is expressed in the stroma of PDAC, while its expression could also be found in neoplastic epithelial cells [6, 14, 15]. Cancer-associated fibroblasts are the main source of FN1 and promote tumor invasion and migration by FN1 assembly [16]. A recent study has also uncovered an anti-metastatic role of fibronectin from tumor cells responding to immunological surveillance of natural killer cells [17]. Moreover, expression of fibronectin in pancreatic tumor cells correlated with poor survival [18]. In a previous proteomic study, we reported that FN1 is an upregulated biomarker in PDAC patients with poor outcome [19]. In this study, we sought to investigate the association of FN1 expression with clinical characteristics and survival of patients with resected PDAC.

## Methods

### Patients and samples

Patients from this study were all diagnosed with PDAC and underwent pancreatectomy at the Department of Surgery, Skåne University Hospital, Lund and Malmö, Sweden. A total of 138 formalin-fixed, paraffin-embedded tissue samples were included. The study period spanned from 1996 to 2017. Hematoxylin and eosin stained tissues from all patients were re-evaluated by our pathologist (A.S.) in accordance with the WHO 2010 classification. As controls, disease-free pancreatic tissues from patients with serous (*n* = 3) or mucinous (*n* = 1) cystadenoma were included. The baseline characteristics of patients with PDAC are presented in Table 1. Ethical approval for this study was granted by the local human ethics committee at Lund University (Ref 2017/320). The study follows the REMARK guidelines where possible [20].

**Table 1:** Baseline characteristics of patients with pancreatic ductal adenocarcinoma (*n* = 138)

Factors	*n* (%)	Median (IQR)	Missing
Age at diagnosis (years)		68.5 (63.0–73.0)	
Gender (female)	73 (52.9)		
Size of primary tumor (cm)		3.0 (2.5–4.0)	
T stage			
- T1	19 (13.8)		
- T2	92 (66.7)		
- T3	26 (18.8)		
- T4	1 (0.7)		
N stage			0.7%
- N0	34 (26.4)		
- N1	52 (37.7)		
- N2	51 (37.0)		
AJCC stage, eighth edition			0.7%
- IA	6 (4.3)		
- IB	20 (14.5)		
- IIA	7 (5.1)		
- IIB	52 (37.7)		
- III	52 (37.7)		
Tumor differentiation			0.7%
- Well	7 (5.1)		
- Moderate	48 (34.8)		
- Poor	78 (56.5)		
- Anaplastic	4 (2.9)		
Positive resection margin (≥R1)	53 (38.4)		
Adjuvant chemotherapy	111 (80.4)		3.6%

*Abbreviations*: *AJCC* American Joint Committee on Cancer

### Tissue microarray

**Fig. 1:**
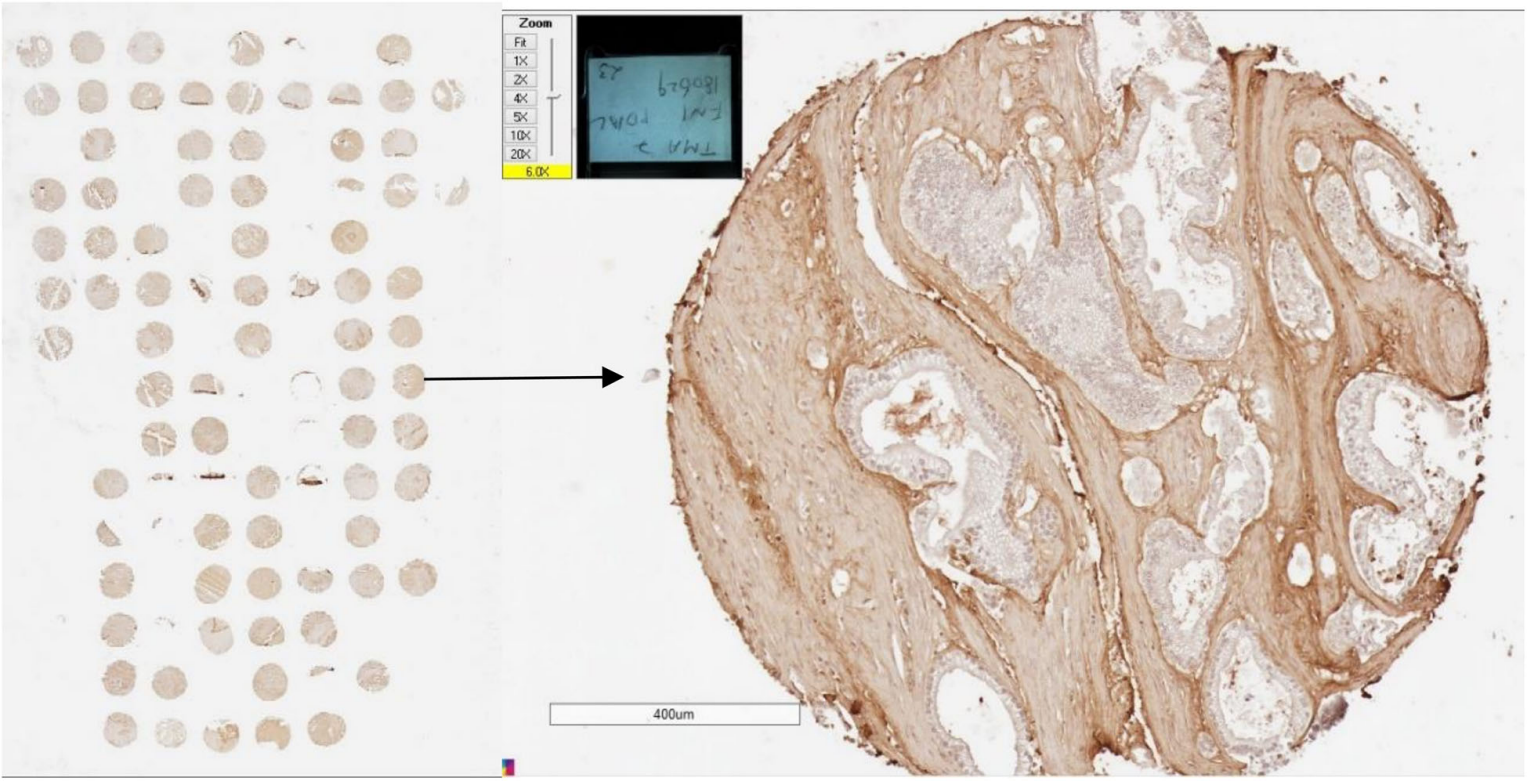
Overview of a tissue microarray slide and overall fibronectin staining

To minimize experimental variability and gain reproducibility, tissue microarray (TMA) technology was applied to the formalin-fixed paraffin-embedded specimens [21]. From each specimen, four sites of cancerous tissues with a diameter of 2 mm were obtained, which were marked by our pathologist (A.S.) and stabilized into paraffin blocks by an automated tissue array device (Minicore® 3, Alphelys, Plaisir, France). The established blocks based on TMA were then sliced into sections with a thickness of 3 μm for further immunohistochemical assessment. Each TMA slide contains around 120 cores, corresponding to samples from 30 patients with 4 replicates. Duplicated TMA slides underwent immunohistochemical staining (Fig. 1).

### Immunohistochemistry

Immunohistochemistry was performed as previously described [22]. Briefly, TMA slides were firstly pre-warmed for 1 h at 60 °C. Secondly, slides were added to EnVision FLEX Target Retrieval Solution low pH (K800521–2, Dako, Glostrup, Denmark) heated to 96 °C for 20 min in an automated PT Link (Dako, Glostrup, Denmark). Then, slides were immersed in phosphate-buffered saline for 5 min, which was repeated twice. Subsequently, slides were immersed into phosphate-buffered saline containing hydrogen peroxide (0.3%) and methanol (1%) for 10 min. The sections were then incubated with 5% goat serum for 1 h at room temperature (RT). After careful removal of the liquid on the slides, successive incubation with avidin and biotin blocking kit (SP-2001, Vector Laboratories, Burlingame, CA, USA) was conducted for 15 min at RT, respectively. Next, the primary antibody, a rabbit recombinant monoclonal FN1 antibody (Abcam, Cambridge, UK; cat no ab2413; dilution 1:4000), was added on the slides. One slide was added with solvent without primary antibody for quality control. After making sure that all tissues were covered by the diluted antibody, samples were preserved in a refrigerator at 4 °C overnight. The next day, biotinylated secondary goat anti-rabbit antibody (BA-1000, dilution 1:200, Vector Laboratories) was applied on the slides at RT for 1 h. To amplify the target antigen signal, an avidin-biotin-peroxidase complex (Vectastain Elite ABC-HRP Kit, PK-6100, Vector Laboratories) was prepared according to the instructions of the manufacturer and used to immerse slides for 30 min at RT. Then, the specimens were covered by chromogen diaminobenzidine (SK-4100, Vector Laboratories) for 5 min, which was followed by deionized water immersion for 5 min. The slides were then immersed in Mayer’s hematoxylin (Histolab, Gothenburg, Sweden) for 30 s and quickly replaced in running tap water for 5 min. Lastly, the slides underwent routine dehydration in alcohol and xylen before mounting by Pertex (Histolab).

### Scoring procedure

The reactivity of the FN1 antibody in samples was evaluated by our pathologist (A.S.), who was blinded to the survival information. The scoring algorithm was modified from Norihiro et al. [23] and takes the proportion of stained cells into consideration, as well as the intensity of the staining. The reactivity was scored in a semi-quantitative manner, which was categorized as negative if less than 10% staining was observed in the stroma; and mild, moderate, or strong based on the intensity if the percentage was > 10%. Low expression was defined as negative and mild reactivity, whereas high expression represented moderate or strong reactivity.

### Statistical analysis

SPSS (IBM. SPSS Statistics for Windows. Version 24.0. Armonk, NY, USA) was used for statistical analysis. Chi-square test or Fisher’s exact test were employed to investigate the association of FN1 expression with clinical characteristics. Kaplan-Meier survival curves were drawn and comparisons were made with the log-rank test. Cox regression proportional hazards models were employed to estimate hazard ratios (HR) according to FN1 expression in both uni- and multivariable analysis, adjusted for age, gender, TNM status, differentiation grade, resection margin, and adjuvant chemotherapy. A two-tailed *P* value < 0.05 was regarded as statistical significance.

## Results

### FN1 expression patterns in pancreatic tissues

**Fig. 2:**
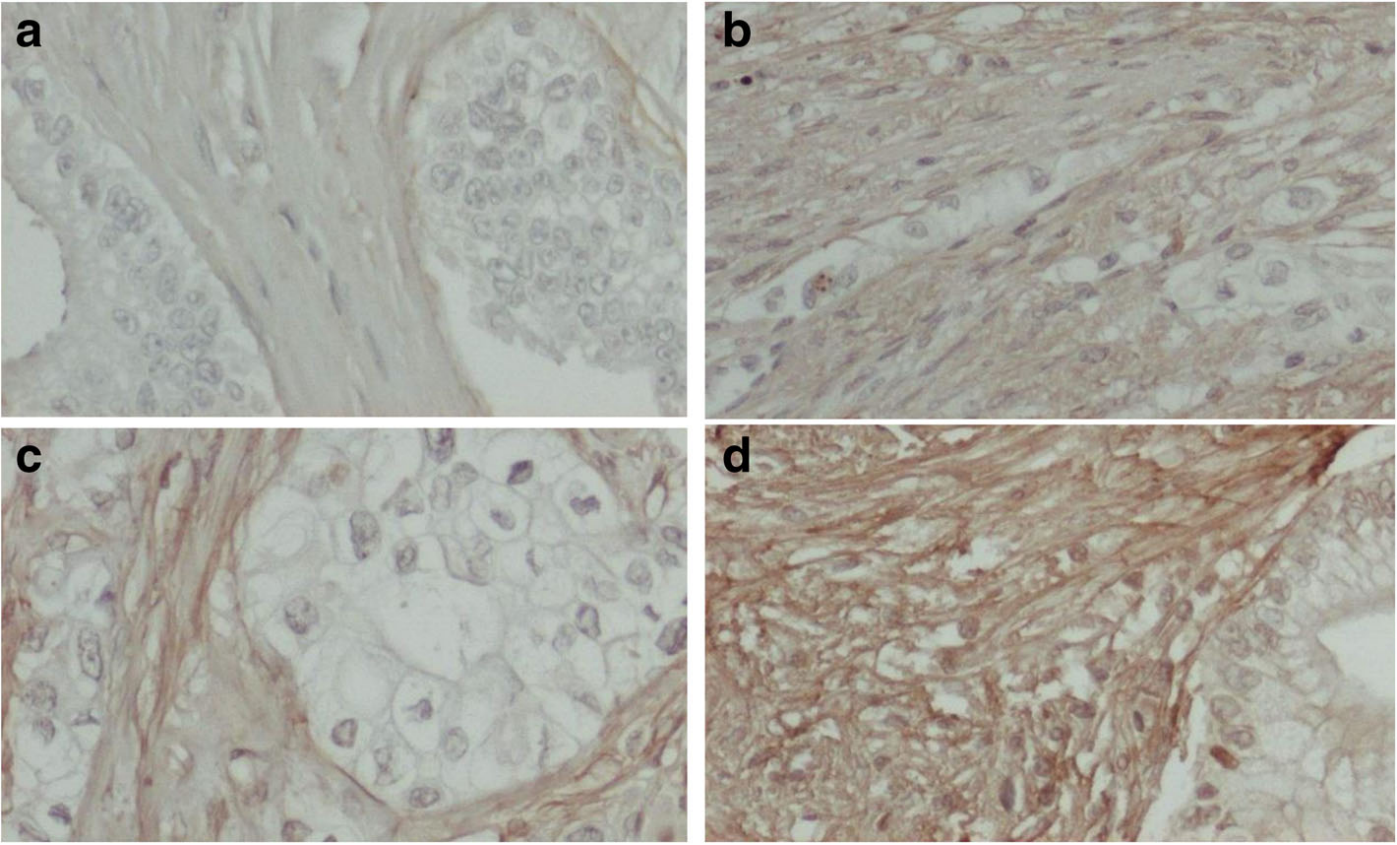
Representative immunohistochemical images with stromal fibronectin expression in pancreatic ductal adenocarcinoma. **a** Negative. **b** Mild. **c** Moderate. **d** Strong

FN1 expression was evaluated in the tumor stroma component, localized to non-malignant fibroblasts and extracellular matrix. The epithelial tumor component was negative. Stromal FN1 expression was negative in 21 (15.3%) of tumors, while 66 (47.8%) tumors had mild FN1 expression, 44 (31.9%) had moderate expression, and 7 (5.1%) had strong FN1 expression. Figure 2 shows representative immunohistochemical images of FN1 expression in PDAC. FN1 was not stained in acinar cells and islets of Langerhans of disease-free control pancreatic tissues (data not shown). The public Human Protein Atlas database also shows absent or minimal expression of FN1 in normal pancreas (https://www.proteinatlas.org/ENSG00000115414-FN1/tissue/pancreas) [24].

### Associations between FN1 expression and clinicopathological characteristics in patients with PDAC

When compared to the low FN1 expression group, the high FN1 expression group had significantly larger tumor size (*P* = 0.002), more advanced T stage (*P* = 0.039) and N stage (*P* = 0.009), and worse AJCC stage (54.0% vs 28.7% with stage III, *P* = 0.003) (Table 2). Furthermore, adjuvant chemotherapy was more common in patients with high FN1 expression as compared to the low expression group (92.0% vs 78.3%, *P* = 0.040). No association was observed between FN1 expression and age, gender, tumor location, tumor differentiation, and resection margin status (all *P* > 0.05).

**Table 2:** Association between fibronectin expression and clinicopathological characteristics

Clinical characteristics	Categories	FN1 low expression (*n*, %)	FN1 high expression (*n*, %)	*P* value
Age	–	69 (63–73)	68 (63–73)	0.618
Gender	Female	46 (52.9)	27 (52.9)	0.994
Tumor size	≦2 cm	21 (24.1)	2 (3.9)	0.002
	> 2 cm	66 (75.9)	49 (96.1)	
T stage	T1	17 (19.5)	2 (3.9)	0.020
	T2	56 (64.4)	36 (70.6)	
	T3	13 (14.9)	13 (25.5)	
	T4	1 (1.1)	0 (0)	
N stage	N0	25 (28.7)	9 (18.0)	0.009
	N1	38 (43.7)	14 (28.0)	
	N2	24 (27.6)	27 (54.0)	
Tumor differentiation	Poor/anaplastic	51 (58.6)	31 (62.0)	0.698
AJCC stage, eighth edition	I-II	62 (71.3)	23 (46.0)	0.003
	III	25 (28.7)	27 (54.0)	
Resection margin	R1	33 (37.9)	20 (39.2)	0.881
Adjuvant chemotherapy	yes	65 (78.3)	46 (92.0)	0.040

*Abbreviations*: *AJCC* American Joint Committee on Cancer

### Association between FN1 expression and survival of patients with PDAC

**Fig. 3:**
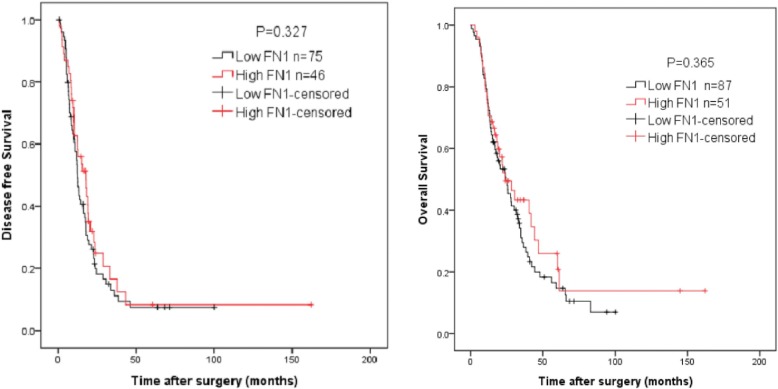
Association between stromal FN1 expression and overall survival and disease-free survival in patients with pancreatic ductal adenocarcinoma (both *P* > 0.05)

Kaplan-Meier analysis showed that there were no differences in either disease-free survival (DFS) or overall survival (OS) when comparing high FN1 expression and low FN1 expression (median DFS 17. 9 vs 12.3 months; median OS 23.8 vs 24.5 months; both *P* > 0.05) (Fig. 3). By using Cox analyses, FN1 expression was not found to be associated with OS or DFS (*P* > 0.05, Tables 3 and 4). On multivariable analysis, only histological grade and resection margin status significantly correlated with DFS or OS. Notably, in our study, there were six patients without complete clinical data (Table 1). Re-analysis with exclusion of these six patients resulted in similar results and the same conclusion.

**Table 3:** Univariable and multivariable Cox survival analyses for disease-free survival

Variables	Univariable analysis			Multivariable analysis		
	HR	95%CI	*P* value	HR	95% CI	*P* value
Age	0.99	0.96–1.01	0.202			
Female gender	0.66	0.45–0.99	0.042			
Tumor size	1.15	0.68–1.94	0.598			
T stage	1.12	0.81–1.55	0.481			
N stage	1.14	0.89–1.45	0.307			
Differentiation grade	1.50	1.07–2.10	0.018	1.54	1.10–2.17	0.012
AJCC stage	1.17	0.78–1.74	0.446			
Resection margin (R1)	1.73	1.14–2.62	0.010	1.84	1.20–2.80	0.005
Adjuvant chemotherapy	1.36	0.77–2.41	0.286			
FN1 expression, high vs low	0.83	0.55–1.24	0.357

**Table 4:** Univariable and multivariable Cox survival analyses for overall survival

Variables	Univariable analysis			Multivariable analysis		
	HR	95%CI	*P* value	HR	95% CI	*P* value
Age	1.00	0.97–1.02	0.737			
Female gender	0.78	0.53–1.16	0.784			
Tumor size	1.08	0.66–1.79	0.758			
T stage	1.13	0.82–1.56	0.472			
N stage	1.14	0.89–1.46	0.294			
Differentiation grade	1.43	1.03–1.97	0.033	1.42	1.02–1.97	0.038
AJCC stage	1.04	0.69–1.56	0.859			
Resection margin (R1)	1.48	0.99–2.22	0.059	1.70	1.12–2.58	0.012
Adjuvant chemotherapy	0.70	0.43–1.15	0.161			
FN1 expression, high vs low	0.82	0.54–1.25	0.366			

## Discussion

PDAC is one of the most stroma-rich cancers. The stroma is composed of non-tumorous cells (such as fibroblasts, pancreatic stellate cells, myofibroblasts, and immune cells), ECM, blood vessels, and soluble proteins including cytokines and growth factors [25]. ECM components are produced by tumor cells and stromal cells and include collagen, FN1, proteoglycans, hyaluronic acid, and SPARC. Collagen, the most abundant ECM component, can bind to the integrin receptor in tumor cells and activate intracellular signaling that induce pro-tumorigenic programs. Proteoglycans consist of core proteins that undergo post-translational glycosylation, which affects cell signaling function [26]. Expression of SPARC has been found to be a strong prognostic factor in patients with PDAC [27, 28]. Due to its overexpression in PDAC and albumin-binding properties, SPARC has been postulated to enhance peritumoral drug delivery of nanoparticle albumin-bound (nab)-paclitaxel [26]. FN1 shares similarities with collagen, as it also preserves binding sites for collagens and supports the role of the latter. In previous experimental studies, it was found that pancreatic cancer cells adhering to FN1 display increased cell proliferation and enhanced chemoresistance [29]. Moreover, cancer-associated fibroblasts assemble FN1 and trigger invasion through integrin-αvβ3 [16].

Our study revealed that expression of FN1 is abundant in the TME of PDAC, while there is a little or minimal expression in normal pancreatic tissue. Stromal FN1 expression was associated with aggressive tumor properties, including larger tumor size, more advanced T stage and N stage, and worse AJCC stage. To our knowledge, this is the first study to report the association of stromal FN1 expression with advanced clinicopathological stage. FN1 is considered to be a biomarker of epithelial–mesenchymal transition (EMT) [18], which has been proposed as a key step for the behavior of tumor metastasis by allowing neoplastic epithelial cells to acquire a more mesenchymal phenotype [30]. It has been reported that EMT status was an important prognostic factor for pancreatic cancer and associated with portal vein invasion and lymph node metastasis, although this study utilized two EMT markers other than FN1 [31].

Previous studies in other malignancies have highlighted the controversial role of FN1 in tumor biology. In glioblastomas, FN1 produced by the tumor cells, facilitate the collective invasion of tumor cell spheroids and significantly enhances tumor growth and angiogenesis [32]. In contrast, using a mouse xenografts model, Liu et al. revealed that silencing of FN1 in human thyroid carcinoma cells exhibited enhanced tumor growth and metastases by upregulation of melanoma-associated antigen [33]. Recently, Glasner and colleagues showed that natural killer cell-mediated IFN-γ production led to the increased expression of FN1 and resulted in decreased metastasis formation in melanoma [17].

In the present study, stromal FN1 expression patterns did not predict survival in PDAC. There is only one previous study on the prognostic impact of FN1 expression in PDAC. In a small series with 34 patients, Javle et al. reported that high expression of FN1 correlated with p-ERK and a worsened survival [18]. Differences between studies may be related to discrepancies in patient cohorts, antibodies, scoring procedures, and interpretations. Furthermore, sample selection bias could exist in our retrospective study. Patients with advanced, non-operable pancreatic cancer were not included in this study. Although FN1 expression-associated clinical characteristics, including tumor size and AJCC stage, were not associated with the survival, they may still confound the role of FN1 in the prognosis of pancreatic cancer. Additional larger studies may be needed to ascertain the potential association of stromal FN1 expression with survival in PDAC.

## Conclusion

The present study showed that stromal FN1expression is associated with larger tumor size, more advanced T stage and N stage, and worse AJCC stage, but not associated with survival in patients with resected PDAC.

## Abbreviations

AJCC: American joint committee on cancer
DFS: Disease-free survival
ECM: Extracellular matrix
EMT: Epithelial–mesenchymal transition
FN1: Fibronectin
OS: Overall survival
PDAC: Pancreatic ductal adenocarcinoma
RT: Room temperature
SPARC: Secreted protein acidic and rich in cysteine
TMA: Tissue microarray
TME: Tumor microenvironment
